# Reply: CD133 expression in different stages of gastric adenocarcinoma

**DOI:** 10.1038/sj.bjc.6605002

**Published:** 2009-03-24

**Authors:** L M Smith, A Nesterova, M C Ryan, S Duniho, M Jonas, M Anderson, M K Sutherland, K L Van Orden, P A Moore, S M Ruben, P J Carter

**Affiliations:** 1 Seattle Genetics, Inc., 21823 30th Drive Southeast, Bothell, WA 98021, USA; 2 Celera, 45 West Gude Drive, Rockville, MD 20850, USA

**Sir,**

The preliminary data presented by Boegl and Prinz reflect CD133 mRNA by RT–PCR rather than protein profiling measurements by IHC as in Smith *et al* (2008). In our study, we used two independent, commercially available antibodies (AC133 and ab5558) to assess the expression of CD133 in formalin-fixed paraffin-embedded samples of various solid tumours, including gastric cancer. The concordance of the data between these antibodies together with numerous positive and negative controls described in Smith *et al* (2008) support the notion that the staining detected in gastric cancer and other samples reflected the presence of the CD133 protein as reported.

In contrast, Boegl and Prinz reported that they were not successful in establishing specific antibody binding for CD133. Unfortunately, no details were provided as to which antibodies were evaluated, nor the methodology used, including controls. We are therefore not able to provide any concrete insights into the discrepancy with our own work. It is interesting that although Boegl and Prinz suggest that the level of expression is reduced in gastric cancer, there appears to be a significant number of outliers that may suggest significant expression in a subpopulation of tumours. From the data in Smith *et al*, the highest IHC staining is in a similar percentage of tumours to the outliers from the Boegl and Prinz experiment.

Lack of agreement between protein-based and mRNA-based profiling measurements is commonplace in the literature (Chen *et al*, 2002; Smith *et al*, 2002; Souchelnytskyi 2002). It appears that CD133 may fall into this category. The RT–PCR approach will potentially miss splice variants and will also not measure changes in protein stability leading to increased levels of protein. In both of these cases, changes in expression can be detected by a protein-based method, such as IHC. In addition, due to heterogeneity of CD133 expression in tumours, RT–PCR can underestimate CD133 expression if no tumour microdissection was applied. Discordance of protein and mRNA is important to note but ultimately protein expression is required for function and determines its potential as a therapeutic target.
